# The Yeast Nucleosome Atlas (YNA) database: an integrative gene mining platform for studying chromatin structure and its regulation in yeast

**DOI:** 10.1186/1471-2164-15-S9-S5

**Published:** 2014-12-08

**Authors:** Po-Cheng Hung, Tzu-Hsien Yang, Hung-Jiun Liaw, Wei-Sheng Wu

**Affiliations:** 1 Department of Electrical Engineering, National Cheng Kung University, No.1 University Road, Tainan City 701, Taiwan; 2 Department of Life Sciences, National Cheng Kung University, No.1 University Road, Tainan City 701, Taiwan

**Keywords:** Chromatin Regulation, Transcription Regulation, Nucleosome Occupancy, Histone Modifications, Regulatory Proteins

## Abstract

**Background:**

Histone modification and remodeling play crucial roles in regulating gene transcription. These post-translational modifications of histones function in a combinatorial fashion and can be recognized by specific histone-binding proteins, thus regulating gene transcription. Therefore, understanding the combinatorial patterns of the histone code is vital to understanding the associated biological processes. However, most of the datasets regarding histone modification and chromatin regulation are scattered across various studies, and no comprehensive search and query tool has yet been made available to retrieve genes bearing specific histone modification patterns and regulatory proteins.

**Description:**

For this reason, we developed the Yeast Nucleosome Atlas database, or the YNA database, which integrates the available experimental data on nucleosome occupancy, histone modifications, the binding occupancy of regulatory proteins, and gene expression data, and provides the genome-wide gene miner to retrieve genes with a specific combination of these chromatin-related datasets. Moreover, the biological significance analyzer, which analyzes the enrichments of histone modifications, binding occupancy, transcription rate, and functionality of the retrieved genes, was constructed to help researchers to gain insight into the correlation among chromatin regulation and transcription.

**Conclusions:**

Compared to previously established genome browsing databases, YNA provides a powerful gene mining and retrieval interface, and is an investigation tool that can assist users to generate testable hypotheses for studying chromatin regulation during transcription. YNA is available online at http://cosbi3.ee.ncku.edu.tw/yna/.

## Background

The eukaryotic genome is packaged into nucleosomes, each of which consists of approximately 147 base pairs of DNA wrapped around a histone octamer and constitutes fundamental unit of the chromatin structure [1,2]. The histone octamer contains H2A, H2B, H3, and H4 proteins which are highly conserved and are subjected to post-translational modifications, including acetylation, methylation, phosphorylation, and ubiquitination [3-5]. Emerging evidence suggests that specifically modified histones function in a combinatorial patterns that can be read by corresponding domains of regulatory proteins and lead to specific cellular events, such as DNA replication and gene transcription [6-8]. In support of this notion, chromatin regulating complexes, such as histone modification enzymes and chromatin remodeling complexes, often contain multiple domains that can bind specifically modified histones. For example, the NuA4 complex, which is a histone acetyltransferase toward H2A and H4, plays an essential role in the transcription of ribosomal genes. Interestingly, it contains chromo, PHD, actin-related, and YEATS domains that interact with methylated H3K36, methylated H3K4, phosphorylated H2A, and acetylated H3, respectively [9]. Another complex SWR1, which contains bromodomains interacting with acetylated H3 and H4, is a chromatin remodeling complex that catalyzes the exchange of histone H2A-H2B dimers with H2AZ-H2B dimers and plays an important role in genome integrity and DNA repair [10]. These multivalent interactions between domains and modified histones have led to the concept of the histone code hypothesis [11-13]. However, the question of how to decipher these combinatorial codes in relation to specific biological events has not been clearly answered. Therefore, there is an urgent need to combine all chromatin related datasets into one platform in order to facilitate biologists for in-depth analyses.

Previous studies have produce several valuable genome-wide datasets of histone modifications, binding occupancy of chromatin-regulating factors, and gene expression in the yeast *Saccharomyces cerevisiae *[14-21]. However, these datasets are scattering across the literatures and researchers are often suffering from searching these fragmentary datasets for further exploration. Despite that a number of databases have been established to perform genome-wide investigation for multiple biological features, there are still no suitable tool for gene mining based on the aspect of chromatin regulation. For example, Saccharomyces Genome Database (SGD) contains comprehensive biological information for *Saccharomyces cerevisiae *[22,23]. SGD also contains the genome browser, GBrowse [24], to display diverse experimental results, including chromatin-regulating features, and to achieve comprehensive genomic overview. Users can view specific regulation of chromatin structure in the specified regions by GBrowse. However, users cannot seek specific gene groups with specifically modified histones or specifically bound factors. Furthermore, it is difficult to correlate the regulations of chromatin structure with transcriptional expression and its binding factors. These information can be extracted only by analyzing the whole genome data through complicated computational procedures. Beside SGD, YeastMine, constructed collaboratively by SGD and the Intermine, is a data search and retrieval tool that incorporates various types of data present in SGD and provides custom query capabilities related to chromosomal features, sequences, protein features, GO annotations, phenotypes, interaction data, expression data, and curated literature [25]. Yet the query engines in YeastMine mainly focus on individual genes and their interactive relationship. Researchers concentrating on chromatin structure cannot find groups of genes sharing the same chromatin regulation pattern from YeastMine directly. Another database, ChromatinDB, provides the query engine to facilitate statistical analysis and visualization of chromatin features based on user-specified gene sets or specific chromosomal regions [26]. The chromatin visualization function on ChromatinDB displays the enrichment of deposited histone modification patterns graphically and further indicates the potential covalent modifications on histones for a given gene sets. However, users need to provide pre-selected gene lists for the analysis. Furthermore, ChromatinDB cannot provide the gene mining function to fetch genes with similar histone modification patterns and associated factors.

Hence we developed the Yeast Nucleosome Atlas database, or the YNA database, to integrate available chromatin related datasets into one platform and to provide a comprehensive query and investigation tool. In YNA, approximately 100 deposited data tracks of nucleosome occupancy, histone modifications, binding occupancy of chromatin-related regulatory proteins are integrated in the database. Besides, YNA also collected global gene expression data and MIPS FunCat functional catalogue for functional analysis. Most importantly, YNA provides the genome-wide gene miner to retrieve genes with user-specified filtering criteria based on the collected datasets. The retrieved genes can be utilized for further analysis. Furthermore, YNA also provides the biological significance analyzer to present the tendency of these chromatin-regulating features for the retrieved genes. This helps users obtain hypotheses to investigate chromatin structure and transcription regulation. Therefore, YNA is a discovery tool that provides the comprehensive investigation for chromatin structure, and can help users propose testable hypotheses for studying chromatin regulation during transcription. YNA is available online at http://cosbi3.ee.ncku.edu.tw/yna/.

## Construction and contents

**Figure 1:**
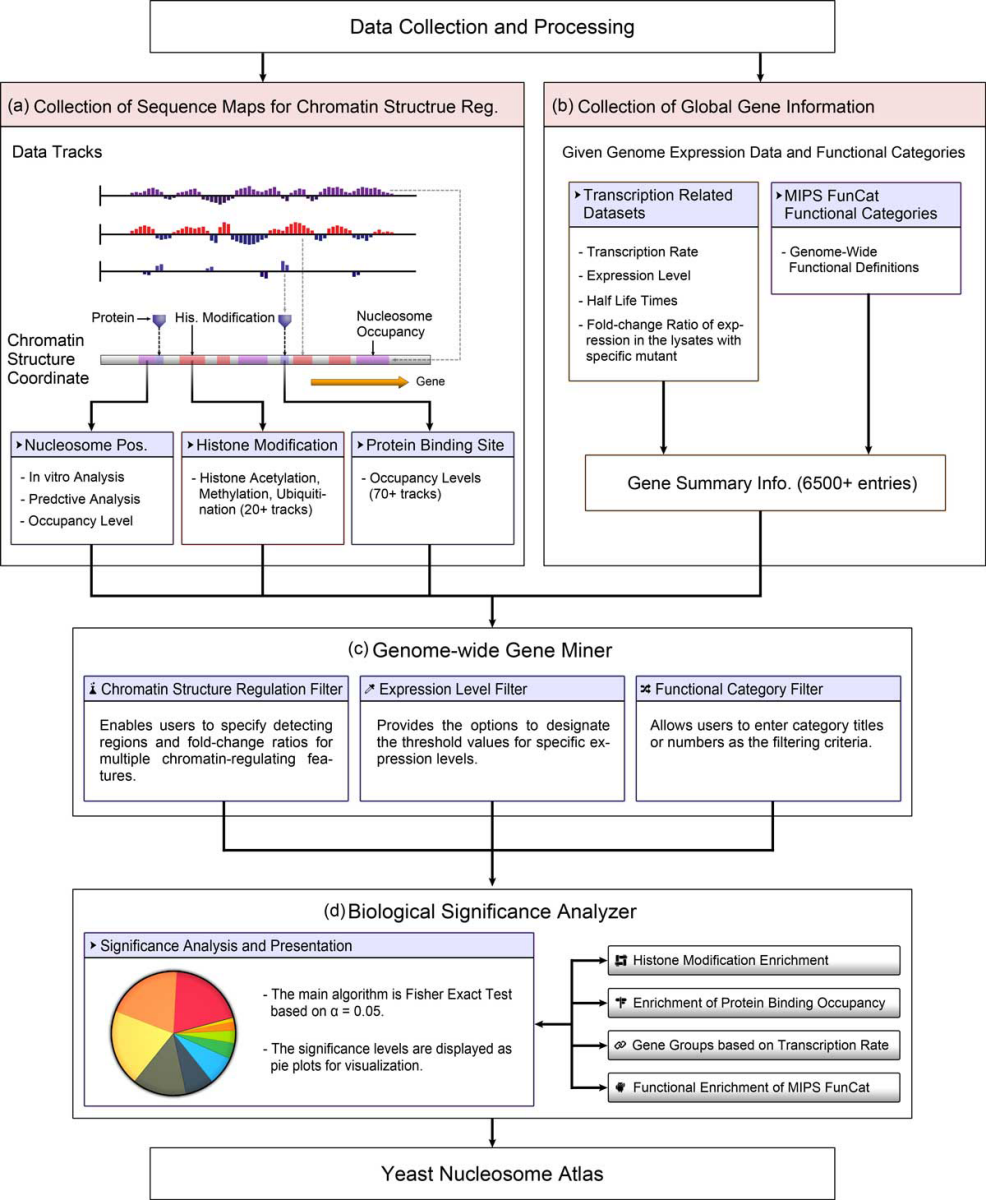
**Construction and architecture of the YNA database**. (a) The data collection of histone modifications, binding occupancy of histones and regulatory proteins. All of these datasets are converted to the corresponding regions of each gene. (b) The data collection of global gene information, which integrates expression data and functional categories. (c) The genome-wide gene miner is the query interface designed for users to retrieve genes that match the various filtering criteria. (d) The biological significance analyzer analyzes the characteristic tendency of these chromatin-regulating features shared by the retrieved genes.

The construction and architecture of YNA are shown in Figure 1. YNA is constructed based on the datasets of chromatin structure regulations, such as histone modifications, histone variants and regulatory protein binding events. These data are reprocessed into genomic sequences of individual genes and eliminated the non-coding sequences. Other datasets like gene expression data and MIPS FunCat functional categories are arranged with the summary information of individual gene. These collected datasets are described in the subsection "Data collection and processing". To acquire the gene query and search interface, the genome-wide gene miner was implemented. We also developed the biological significance analyzer for further analysis of the retrieved genes. These two features are described in the subsection of "Genome-wide gene miner" and "Biological significance analyzer", respectively.

### Data collection and processing

In YNA, all collected datasets can be classified into 8 classes based on the properties. These 8 classes are the genome summary information, the nucleosome positioning data, the histone modification data, the histone variant data, the binding occupancy data of regulatory proteins, the gene expression data, the expression data of mutant cells, and the data of MIPS functional category.

#### Yeast gene collections

The yeast genome summary was retrieved from Saccharomyces Genome Database (SGD) [22,23]. The gene summary includes the systematic name, gene alias, gene description and coordinates of coding regions for about 6500 genes.

#### Nucleosome positioning data

Three datasets of genome-wide nucleosome organization maps are included in the YNA database [14-16]. In YNA, the datasets of nucleosome organization are displayed as sequence coordinate charts with corresponding predicted occupancy scores or normalized detection values of nucleosome occupancy.

#### Histone modification data

Post-translational modifications of histones play crucial roles in altering chromatin structure and creating marks for the binding sites of specific chromatin regulatory proteins, thereby regulating genomic expression behaviors [5]. In YNA, approximately 20 different data tracks related to histone modification were collected. These data present the modification states in the formats of enrichment levels for the whole genome. The main composition of histone modification patterns employed in YNA are histone acetylation (acetyl-lysine 4 of histone H3 (H3K4ac), acetyl-lysine 9 of histone H3 (H3K9ac), acetyl-lysine 14 of histone H3 (H3K14ac), and histone H4 acetylation (H4ac)), methylation (monomethyl-lysine 4 of histone H3 (H3K4me), dimethyl-lysine 4 of histone H3 (H3K4me2), trimethyl-lysine 4 of histone H3 (H3K4me3), trimethyl- lysine 36 of histone H3 (H3K36me3), dimethyl-lysine 79 of histone H3 (H3K79me2), and trimethyl-lysine 79 of histone H3 (H3K79me3)) and ubiquitination (monoubiquitination of histone H2B on lysine 123 (H2BK123ub)) [17-20]. In addition, data tracks of histone modifications in mutant cells, such as *set1Δ, ubp8Δ*, and *ubp10Δ*, are also included.

#### Data of binding occupancy of regulatory proteins

In eukaryotes, transcription is regulated by hundreds of proteins. These proteins consist of sequence-specific DNA-binding proteins, chromatin regulators, elongation regulators and transcription factors [27,28]. We gathered the data of binding occupancy of 73 proteins, which belong to proteins of chromatin remodeling and histone modifications [21]. In YNA, the data of binding occupancy of regulatory proteins are displayed as the coordinate charts with fold-change ratios over the backgrounds. The fold-change ratios indicate the binding enrichments of regulatory proteins in the selected regions.

#### H2A.Z histone variant data

H2A.Z, the histone variant of H2A, has been widely studied and is thought to regulate several biological processes. The global H2A.Z map is also included in the YNA database [29].

#### Gene expression data

The expression data of each gene are collected in YNA [30]. The adopted dataset has three types of expression data under YPD growth condition. The three expression data are the transcription rate of mRNA per hour, the expression level calculated from mRNA copies per cell, and the half life time of mRNA, respectively.

#### Data of expression profiles of mutant cells

The expression data of mutant cells, including *arp8Δ, ino80Δ, set1Δ*, and H3K4R (the replacement of lysine 4 with arginine in histone H3), are also collected in YNA [17,31]. Ino80 and Arp8 are two subunits of INO80 complex, which is an ATP-dependent chromatin remodeler. Mutations in the *ino80Δ *and *arp8Δ *genes are defective in DNA double-strand break repair and in gene transcription. The dataset includes the fold-change of expression levels in the *ino80Δ *and *arp8Δ *mutant cells, compared to wild type cells.

#### Data of MIPS FunCat functional annotation

MIPS FunCat (MIPS Functional Catalogue) [32,33] is a functional annotation scheme with systematic classification and clear definitions for the gene functions. FunCat consists of 28 main categories, which are hierarchically structured and contain multiple child nodes representing distinct functions in several levels. In YNA, we retained about 500 FunCat catalogue nodes with entries matching in yeast genes and integrated them into the gene summary information.

### Genome-wide gene miner

We implemented the genome-wide gene miner in YNA to retrieve genes that match the filtering criteria. The genome-wide miner has three main filter set, which are chromatin structure regulation filter, expression level filter, and functional category filter, respectively. The three filters can be used with combination.

#### Chromatin structure regulation filter

The main purpose of chromatin structure regulation filter is to query for genes that match specific chromatin-regulating features, such as histone modifications, histone variants and binding occupancy of regulatory proteins, in promoters or coding regions. In this filter set, users are able to specify the threshold ratios relative to the background. The region options consist of (I) coding region of genes, (II) promoter region, which is defined between 500 bp upstream and 100 bp downstream from the gene start site, (III) both coding and promoter regions, and (IV) either of the two regions, separately. To achieve better query performance, we reprocessed all data from original sequence-formatted rows into individual gene regions and eliminated those non-coding sequences to abandon superfluous noises. While executing the query process, YNA globally traverses all sample values inside targeted regions from the rearranged data to filter out those target genes matching the defined criteria.

#### Expression level filter

The format of expression data deposited in YNA are measured by each gene and can be roughly divided into two types. The first type is quantified transcriptional property such as transcription rate and the counts of mRNA copies per cell, which directly deliver the information of gene's capability [30]. The other one is the difference ratio of expression level under specific induced mutants (*ino80Δ, arp8Δ, set1Δ*, H3K4R) comparing to wild types [17,31]. Expression level filter operates with these datasets based on numeric filtering, where users can define the threshold values and mathematical operators for each expression feature as the filtering criterion in this filter set.

#### Functional category filter

The functional category filter provides the interface to fetch those genes that match the MIPS Functional Catalogue. In this filter set, users are able to specify keywords or category numbers to retrieve those genes with matching functional categories.

### Biological significance analyzer

The genome-wide gene miner provides the interface for users to extract genes that match the query criteria. To gain insight into the correlation between chromatin structure and transcription regulation of the retrieved genes, we implemented the biological significance analyzer along with the query results. The analyzer provides the enrichment analyses in four aspects: (I) the enrichment of histone modifications, which calculates the significance levels of the specific histone modification patterns in promoters and coding regions; (II) the enrichment of binding occupancy of regulatory proteins, which calculates the significance levels of regulatory protein binding events in promoters and coding regions; (III) mRNA transcription rate, which evaluates the significance levels of the transcription rate of the retrieved genes. The transcription rate is divided into 5 groups: "greater than 50 mRNA/hr", "16 - 50 mRNA/hr", "4 - 16 mRNA/hr", "1 - 4 mRNA/hr", and "less than 1 mRNA/hr"; and (IV) the functional enrichment of MIPS functional categories of the retrieved genes.

The main algorithm to analyze the significance is based on Fisher Exact Test. Fisher Exact Test is used for analyzing the statistical significance of the proportion representing some categorized data subsets, such as those classified feature patterns mentioned above, within a given gene list relative to the genomic abundance ratio with the same property [34]. YNA adopts a statistic confidence level of α = 0.05. To acquire more visual conception, the calculated *p*-values in the four analyses are converted into positive correlated scores by taking the negative logarithm and are then displayed as pie plots.

The biological significance analyzer only displays those significantly enriched features (we adopted a two times fold-change ratio as the enrichment threshold for all features). The resulting analysis demonstrates the tendency of these features shared by the retrieved genes. Moreover, this provides hypotheses for the combinatorial effects of those significant chromatin-regulating features for downstream analysis.

## Utility and discussions

### Database implementation

The construction of YNA is based on CodeIgniter PHP Framework and MySQL database management system. The design layout of website interface was extended from YAML CSS Framework and jQWidgets. To improve the browse experience and performance, we developed vast JavaScript (ECMAScript) applications. Furthermore, all query operations in YNA are achieved in REST (Representational state transfer) architectural style. The sequence maps of chromatin structure regulations are presented as static images, which are produced by MatplotLib, a library of the Python scripting language.

### Database interface

**Figure 2:**
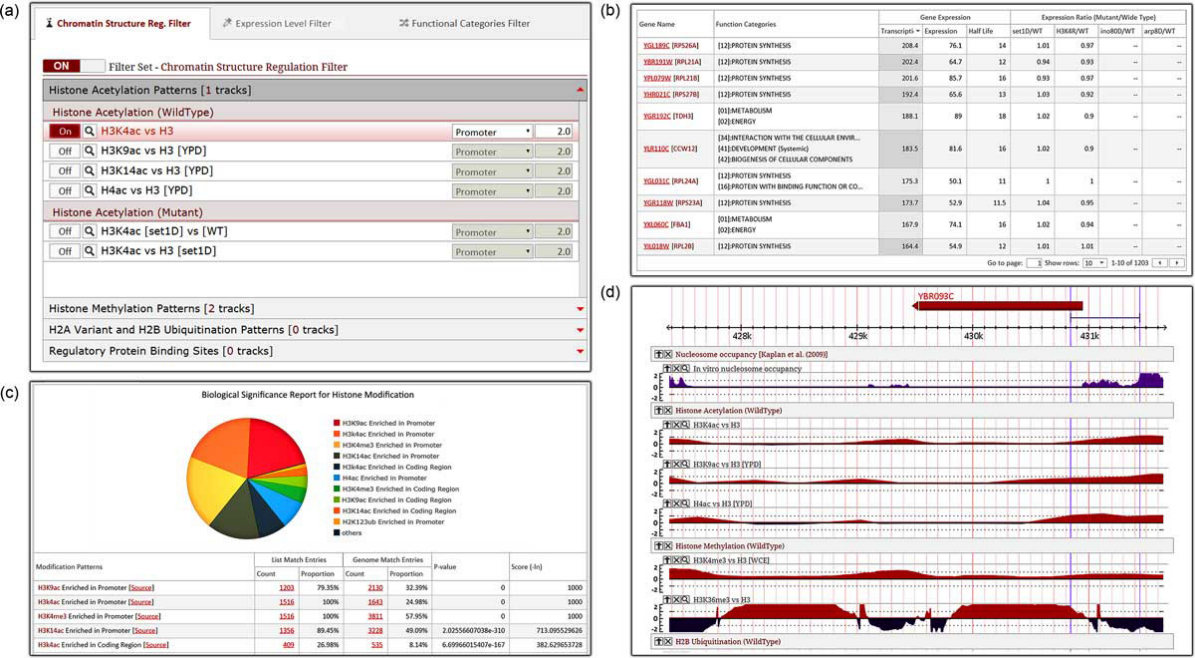
**The functional panoramic view of YNA database**. (a) The snapshots of genome-wide gene miner. (b) The gene list is retrieved through the genome-wide gene miner. The table information includes the gene name, functional categories, expression levels, and expression differences between wild type and mutant cells. (c) The biological significance analyzer provides the significance analysis of the retrieved genes and displays the results as pie plots. (d) The sequence maps of histone modifications in the specified gene region.

The YNA database provides three main functional modes for users to investigate the regulations for chromatin structure: (I) The query mode contains the manipulation panel of the genome-wide gene miner and the gene query engine (Figure 2a); (II) The list mode presents the list of retrieved genes that match the query criteria (Figure 2b). The biological significance analyzer displays the analysis results of the retrieved genes as pie plots (Figure 2c); (III) The detail mode exhibits the basic description and the chromatin-related patterns of the specified gene (Figure 2d). The query mode is set up in the query page, and the other two modes are integrated in the result page. A functional panoramic view of the YNA database is shown in Figure 2.

The genome-wide gene miner is the main function in the query mode. Users are able to specify filtering criteria based on the three filter sets, i.e. chromatin structure regulation filter, expression level filter, and functional category filter, and then execute the query to retrieve genes that match the filtering criteria (Figure 2a). The chromatin structure regulation filter enables users to specify coding or promoter regions and fold-change ratios of specific histone modifications and binding occupancy of regulatory proteins. The expression level filter provides the options for users to specify threshold values of expression levels. The functional category filter allows users to enter MIPS FunCat category keywords or numbers to search genes that match the search criteria. YNA also provides query defaults, which are pre-defined filter settings, so that users can easily get started. Furthermore, we also constructed the traditional gene query engine in the query mode, which enables users to search individual genes by their systematic names or standard names.

In the list mode, the retrieved genes are displayed in the table. The genes are also analyzed through the biological significance analyzer in terms of four aspects: histone modifications, binding occupancy of regulatory proteins, transcription rates, and functional categories. All of the displayed table contents and charts can be downloaded in multiple file types (Excel table, CSV table, XML format and JSON format). The interface of the list table and biological significance analyzer mounted in list mode can be viewed in Figures 2b and Figure 2c.

In the detail mode, the summary of the specifically selected gene is exhibited. The summary includes the information of the specified gene and sequence maps of multiple chromatin-related datasets, including nucleosome positioning, histone modifications and the binding occupancy of regulatory proteins, near the gene region. These sequence maps are shown in log2 fold-change ratios to represent the enrichment or depletion of specific chromatin regulation patterns (Figure 2d).

All processed plain text files of chromatin-related datasets are annotated with their sources and are available for downloading in the statistics page. We also provided a detailed tutorial page so that users can fully utilize functions in YNA.

### Case study

To demonstrate the usability of YNA, we query genes with specific filtering criteria in the genome-wide gene miner. The retrieved genes are further analyzed in the biological significance analyzer to gain insight into information of the retrieved genes. In *S. cerevisiae*, histone acetylation and methylation are widely studied and are thought to regulate gene transcription [8]. Hence we took following two examples describing the functionality of YNA.

#### Acetyl-lysine 4 of histone H3 (H3K4ac) and trimethyl-lysine 4 of histone H3 (H3K4me3)

**Figure 3:**
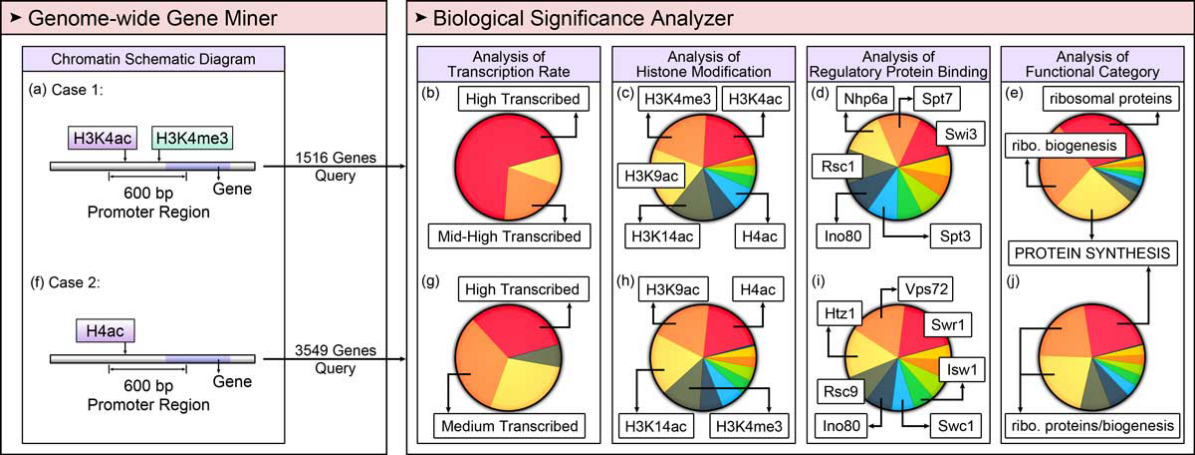
**Case studies using the YNA database**. (a) A combined search of H3K4ac and H3K4me3 in the promoter region in the genome-wide gene miner of the YNA, which leads to the retrieval of 1516 genes that match the query criteria. The retrieved genes were further analyzed using the biological significance analyzer, which displays the enrichments of the histone modifications (b), the enrichments of the regulatory protein binding occupancy (c), the transcription rate in 5 groups (d), and the enrichments of the functional categories (e) of the retrieved genes. (f) Another search of H4ac enriched in the promoter region, which results in the retrieval of 3549 genes that match the query criteria. (g - j) The biological significance analyzer analyzes these retrieved genes and provides the enrichment analyses in the four aspects.

Histone modifications, such as the modifications of H3K4ac and H3K4me3 enriched in promoters, have long been known to be associated with actively transcribed genes. Therefore, we set the chromatin structure regulation filter of the genome-wide gene miner, such that both H3K4ac and H3K4me3 must have fold-changes equal to or greater than 2 folds in promoters. 1516 matched genes were retrieved (Figure 3a). Consistent with previously published results, we discovered that these genes are transcribed either at a high level (greater than 50 mRNA/hr) or at a mid-high level (16 - 50 mRNA/hr), with *p*-values of 10^-93 ^and 10^-28^, respectively, through the biological significance analyzer (Figure 3b) [17,20]. In addition, the acetyl-K9 of histone H3 (H3K9ac), the acetyl-K14 of histone H3 (H3K14ac), and H4 acetylation are also enriched in promoters of the 1516 genes (Figure 3c). Consistent with previously published results, H3K9ac, H3K14ac, and H4ac were also shown to be associated with highly transcribed genes [20,27,35].

Furthermore, we discovered that several regulatory proteins, including Swi3, Rsc1, and Ino80, are associated with promoters of these genes with *p*-values of 10^-56^, 10^-43^, and 10^-42^, respectively (Figure 3d) [36]. These regulatory proteins were known to be involved in chromatin remodeling. Finally, applying functional category analysis to the 1516 genes, most were found to participate in ribosomal protein synthesis and metabolism related enzyme synthesis, which are necessary for the translational machinery (Figure 3e) [8].

#### Histone H4 acetylation (H4ac)

Histone H4 acetylation enriched in promoters, has long been considered to associate with transcription. Therefore, we set the filtering criterion in the genome-wide gene miner that H4ac has fold-changes equal or greater than 2 fold in promoters, and we retrieved 3549 matching genes after the query (Figure 3f). In the biological significance analyzer, we discovered that these genes are transcribed either at high level (greater than 50 mRNA/hr) or at medium level (4-16 mRNA/hr) with *p*-values of 10^-18^, and this result is consistent with previously published results (Figure 3g) [5]. Besides, some modification patterns, such as H3K9ac, H3K14ac, and H3K4me3, are also enriched in promoters of these genes with *p*-value less than 10^-277 ^(Figure 3h). This suggested the potential that H3K9ac, H3K14ac, and H3K4me3 are also associated with highly transcribed genes, which are also supported by previous studies [20,27].

Furthermore, several regulatory proteins, including Swr1, Vps72, Rvb1, and Esa1, were found to be associated with promoters of these genes with *p*-value less than 10^-12 ^(Figure 3i). Swr1, Vps72, and Rvb1 belong to SWR1 complex, whereas Esa1 belongs to NuA4 complex. NuA4 is a histone acetyltransferase toward H2A and H4. Therefore, the enrichment of Esa1 is consistent with the enrichment of H4ac [8]. Interestingly, NuA4 complex is highly conserved from yeast to human. TIP60 is human homolog of yeast NuA4 complex. TIP60 corresponds to a near-exact merge of yeast NuA4 and SWR1 complexes [37,38]. The finding that Swr1, Vps72, and Rvb1 are also enriched in promoter as the enrichment of H4ac, suggesting that NuA4 and SWR complexes might work together to regulate transcription in yeast [39].

#### Summary

The two case studies demonstrated that histone acetylation (H3K4ac and H4ac) and methylation (H3K4me3) are highly correlated with transcription activities [8]. Moreover, the biological significance analyzer provides testable hypotheses, which have been validated in the literatures. Thus, YNA provides a platform for researchers to investigate chromatin regulations, and to generate testable hypotheses that can be further verified in experiments.

### Issues related to YNA

YNA deposits genome-wide chromatic-regulation datasets for elucidating the transcriptional mechanisms caused by alterations on chromatin structure. However, these datasets came from several studies, and the experimental procedures may vary from one laboratory to another, thus resulting in potential systematic biases. In systems biology, a cellular system is perturbed and measured by high-throughput technologies [40]. Hence, the integration of different high-throughput assays, which were probably performed on different cell states, suffers from the systematic bias and unpredictable noises [41,42]. Since this type of systematic bias is still unavoidable [41], some crucial points are worthy of attention when browsing these regulating identification of chromatin structure.

In YNA, we collected the datasets of histone modifications, protein binding occupancy and expression data from multiple studies. In these data, the yeast strains used for chromatin immunoprecipitation or expression profiling experiments are derived either from yeast *Saccharomyces cerevisiae *strain S288c or W303, which share very similar genetic background and culture condition (cells growing to log-phase at 30°C in YPD). Hence the systematic bias of condition variation in the data integration of YNA is not severe. Besides, to provide more detailed information for each dataset, we have marked out the experimental environments and the assay resolutions for which the high-throughput experiments were performed.

On the other hand, it is well known that post-translational modifications of histones, particularly acetylation and methylation, play wide-spread roles in transcription regulations. But nowadays the genome-wide sequence maps of histone modification are still not completely available for all possible combinations of those features in all different cellular conditions [5,8]. Furthermore, studies focusing on histone modifications so far have examined modifications in cells that are cycling asynchronously and are growing in rich media. Therefore, these datasets are simply a snapshot of histone modifications at one point during transcription [5]. This fact limits the possibility that histone modification status might change in response to environmental stresses. In the current version of YNA, we aimed to develop the visual and analysis tool for genome-wide chromatin structure regulations. And all of these analyses are based on steady-state with respect to gene expression. We will incorporate more comprehensive data collection as the updating plan when new datasets related to histone modifications in different cellular conditions are published in the future.

### Availability and requirements

YNA is available at http://cosbi3.ee.ncku.edu.tw/yna/. We recommend users to browse YNA by Google Chrome. Other modern browsers such as Microsoft IE9, Apple Safari and Mozilla Firefox 21 also perform well in YNA. JavaScript is required in YNA functionality and should be enabled.

## Conclusions

In this study, we developed the YNA database, which integrates a number of scattered datasets of histone modifications, binding occupancy of regulatory proteins, gene expression data, and functional categories. Most importantly, YNA provides the genome-wide gene miner that allows user to retrieve genes based on combinatorial filtering criteria of specific histone modification patterns, expression levels, and functional categories. These retrieved genes can be further analyzed using the biological significance analyzer for the enrichments of specific chromatin-related features. Therefore, YNA is an investigation and discovery tool that can be used to mine proper targeted genes and to propose testable hypotheses for further chromatin studies. YNA will be updated regularly to keep with the latest chromatin-regulation datasets.

## List of abbreviations

SGD, Saccharomyces Genome Database; MIPS, Munich Information Center for Protein Sequence; FunCat, Functional Catalogue; YNA, Yeast Nucleosome Atlas database.

## Competing interests

The authors declare that they have no competing interests.

## Authors' contributions

PCH processed collected datasets and constructed YNA website. PCH and THY wrote the manuscript. HJL provided essential guidance and inspected biological issues. HJL and WSW conceived the research topics. PCH, THY and HJL proofread the manuscript. All authors have read and approved the final manuscript.

## Declarations

The publication of this supplement was funded by the Aim for the Top University Project to the National Cheng Kung University.

This article has been published as part of *BMC Genomics *Volume 15 Supplement 9, 2014: Thirteenth International Conference on Bioinformatics (InCoB2014): Computational Biology. The full contents of the supplement are available online at http://www.biomedcentral.com/bmcgenomics/supplements/15/S9.
